# New insights in efficacy of different ERT dosages in Fabry disease: Switch and switch-back studies data following agalsidase beta shortage. Update of systematic review

**DOI:** 10.1016/j.gimo.2023.100805

**Published:** 2023-03-23

**Authors:** Eleonora Riccio, Carlo Garofalo, Ivana Capuano, Pasquale Buonanno, Guido Iaccarino, Teodolinda Di Risi, Massimo Imbriaco, Federica Riccio Cuomo, Antonio Pisani

**Affiliations:** 1 Institute for Biomedical Research and Innovation, National Research Council of Italy (IRIB CNR), Palermo, Italy; 2 Division of Nephrology, University of Campania “Luigi Vanvitelli,” Naples, Italy; 3 Chair of Nephrology, Department of Public Health, University Federico II of Naples, Naples, Italy; 4 Department of Neurosciences, Reproductive and Odontostomatological Sciences, University of Naples Federico II, Naples, Italy; 5 Department of Advanced Biomedical Sciences, University of Naples Federico II, Naples, Italy; 6 CEINGE - Biotecnologie Avanzate, Via Gaetano Salvatore, Naples, Italy; 7 Department of Radiology, Federico II University of Naples, Naples, Italy; 8 Universisad Anáhuac Puebla, San Andrés Cholula, Puebla, México

**Keywords:** Agalsidase alfa, Agalsidase beta, Enzymatic replacement therapy, Fabry disease, Switch

## Abstract

In 2016, a systematic review and a meta-analysis of existing data on the effects of switch from agalsidase beta to alfa in patients with Fabry disease showed that the switch was well tolerated and associated with stable disease progression. However, additional evidence that supports the need for an update of the review on the long-term effects of switching to agalsidase alfa, with a mention on the effects of reswitch to agalsidase beta, has emerged. Relevant papers were identified on PubMed, Cochrane, ISI Web, and Scopus databases from September 2015 to December 2021. Analyzed parameters were clinical events, changes in organ function or structure, disease related symptoms, lyso-globotriasolylceramide 3 (lyso-Gb3) plasma levels, presence of antidrug antibodies, and adverse effects. In total, 15 publications were evaluated, with a total of 353 subjects. The results of the review confirmed some points of the previous analysis with some new important information. After the switch from agalsidase beta to alfa, an increased number of clinical events, a significant loss of renal function, and an increase in lyso-Gb3 levels were reported; conversely, lyso-Gb3 levels decreased after the switch from agalsidase alfa to beta. The results confirm the importance of dose and recommend that patients be monitored through intensified surveillance, including lyso-Gb3 levels every 6 months.

## Introduction

Fabry disease (FD) is a rare, progressive multisystemic disorder, caused by deficiency of the lysosomal enzyme alpha-galactosidase A.^1^^,^^2^ The current treatment options for FD include intravenous enzyme replacement therapy (ERT)^3^^,^^4^ and, more recently, an oral chaperone therapy.^5^^,^^6^ Two preparations for ERT have been commercially available in Europe for more than 20 years: agalsidase alfa (Replagal, Takeda) and beta (Fabrazyme, Sanofi). Although these formulations are biochemically and structurally very similar,^7^ agalsidase beta contains oligosaccharides with a higher content of sialic acid and more mannose-6-phosphate than agalsidase alfa. Because the entry of recombinant agalsidase into cells is mediated by mannose-6-phosphate receptors, a greater absorption of agalsidase beta than alfa has been showed both in vitro and in vivo.^7^^,^^8^ Moreover, they are produced using different methods and approved at different dosages^2^; therefore, a clinical discussion about the most effective dose of enzyme was unavoidable. The literature showed that both preparations are safe and effective in patients with FD,^9^^,^^10^ but very few data directly compare the clinical effects of the 2 drugs.

The worldwide shortage of agalsidase beta supply (from June 2009 to January 2012) because of a viral contamination in the manufacturing process of Fabrazyme resulted in a change of therapy from agalsidase beta (1.0 mg/kg e.o.w.) to agalsidase alfa (0.2 mg/kg e.o.w.),^11^, ^12^, ^13^ offering the possibility to compare, although indirectly, the effects of the 2 drugs on the progression of the disease. Because single studies reporting the effect of switch are limited and nonconclusive, in 2016, a meta-analysis that included a systematic review of 9 studies (217 patients) published from July 2009 to September 2015 was performed.^14^ The results showed only marginal differences in most of the evaluated parameters and that the switch to agalsidase alfa was well tolerated and associated with stable clinical conditions. However, it was impossible to determine whether clinical stability and efficacy are maintained in the long term for the short follow-up period.

New long-term evidence has emerged since the last review; a re-evaluation of the existing literature with an update of the systematic review was performed.

The aim of this paper was to update the findings reported in the previous review to determine whether more recently published literature provided further evidences about the effects of switch of ERT in FD and confirm the comparable effects of the 2 drugs.

## Materials and Methods

### Data sources and study selection

An electronic search of 4 databases (PubMed, Cochrane, ISI Web of Science, and Scopus) was independently conducted by 2 researchers. The terms “Fabry disease”, “agalsidase alfa”, “Replagal”, “agalsidase beta”, or “Fabrazyme” were searched and crossed with “switch”, “switching”, or “shortage”. Because the search limits of the previous systematic review were dated up to September 2015, data limits of this review were September 2015 to December 2021.^15^ Then, data of identified studies were analyzed with the publications included in our previous review.^14^^,^^16^, ^17^, ^18^, ^19^, ^20^, ^21^, ^22^, ^23^, ^24^

### Eligibility criteria

We included studies reporting outcomes after switch from agalsidase beta to agalsidase alfa in patients with FD, whereas the following exclusion criteria were applied: (1) duplicate publication; (2) case reports, comments, expert opinions, editorials, and commentaries; (3) studies analyzing the same sample; and (4) lack of data on outcomes after switch.

Furthermore, data after switch-back from agalsidase alfa to beta were also included in the systematic review.

### Data extraction

Two reviewers independently extracted data from included studies, and any discrepancy was resolved through consensus. The investigators of included studies were contacted for missing data.

### Outcome measures

The following FD-related progression parameters were analyzed.

#### Clinical events

Clinical events included (1) death; (2) cardiac events: symptomatic arrhythmia requiring implantable cardioverter-defibrillator or pacemaker implantation, myocardial infarction, coronary artery bypass graft, or percutaneous transluminal coronary angioplasty; (3) renal events: progression of chronic kidney disease to stage 5, ie, estimated glomerular filtration rate (eGFR) of <15 mL/min per 1.73 m^2^ (with decrease of eGFR ≥30%) needing kidney transplantation or dialysis; and (4) neurologic events: stroke or transient ischemic attack.

#### Changes in organ function or structure

Changes in organ function were investigated at cardiac, renal, and neurologic level. At cardiac level, echocardiographic (thickness of cardiac structures, left ventricular volume, systolic and diastolic function, and heart rate), electrocardiographic, and magnetic resonance imaging data were evaluated. Renal function was evaluated through changes in eGFR and urine albumin-to-creatinine ratio in spot samples. Neurologic modifications were investigated on the basis of clinical examination, interview regarding stroke or stroke-like symptoms, and quantitative sensory testing for cold detection threshold assessment.

#### Changes in FD-related symptoms and questionnaires

These symptoms included: gastrointestinal pain; diarrhea; hypohidrosis or anhidrosis; tinnitus; acroparesthesia, chronic pain, and pain crises as assessed by the Graded Chronic Pain Score and the Neuropathic Pain Symptom Inventory score, the Brief Pain Inventory questionnaire,^25^ and the McGill pain questionnaire^26^; fatigue; the Mainz Severity Score Index (MSSI)^27^; and quality of life (QoL) as determined by the Short Form 36 (SF-36)^28^ or EuroQol dimensions.^29^

#### Changes in lyso-Gb3 plasma concentration

##### Antibodies

Presence of antidrug antibodies (ADA) and neutralizing ADA was not discussed in our previous review but was now investigated and included in the qualitative analysis.^30^^,^^31^

##### Adverse effects

The considered adverse effects (AEs) were dyspnea, hypertension, gastrointestinal symptoms, rigors, temperature change sensation, fever, headache, rhinitis, flushing, and pruritus.

Owing to the lack of comparability among studies in some outcomes and unavailability of some data, we did not perform a quantitative analysis. Furthermore, only 1 study could be added to our previous meta-analysis making the quantitative analysis useless.

## Results

**Figure 1:**
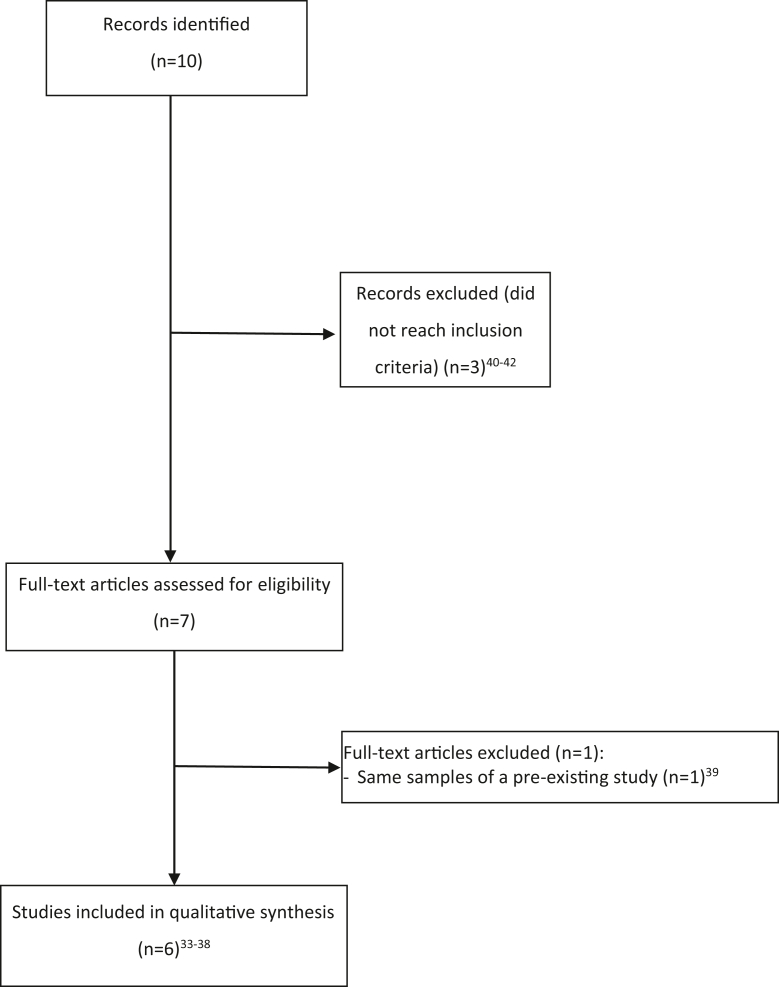
Preferred Reporting Items for Systematic Reviews and Meta-Analyses flow diagram of the search strategy.

The updated electronic literature research (September 2015-December 2021) resulted in 10 papers.^32^, ^33^, ^34^, ^35^, ^36^, ^37^, ^38^, ^39^, ^40^, ^41^ After reading the full texts, 3 articles were excluded,^37^, ^38^, ^39^ and only 7 publications^32^, ^33^, ^34^, ^35^, ^36^, ^37^, ^38^ fulfilled our search strategy. However, the study by Krämer et al^38^ was excluded because it was reanalyzed by Lenders et al^34^ after a longer follow-up period, and finally, 6 studies were included in the qualitative synthesis.^32^, ^33^, ^34^, ^35^, ^36^, ^37^ Our previous systematic review identified 9 papers,^16^, ^17^, ^18^, ^19^, ^20^, ^21^, ^22^, ^23^, ^24^ which were included in this narrative synthesis review in combination with the new 6 publications. Thus, an overall of 15 publications were included in this updated review^17^, ^18^, ^19^, ^20^, ^21^, ^22^, ^23^, ^24^, ^25^, ^33^, ^34^, ^35^, ^36^, ^37^, ^38^, ^39^ (Figure 1).

Included papers reported data on an overall population of 353 patients with FD (217 patients already included in the previous review and 136 from new papers). The main characteristics of the included studies are reported in Table 1. The studies showed a wide variability in the characteristics of patients, duration of treatment period, and outcomes. Some of the new included studies also evaluated effects of switch from agalsidase alfa to beta.^33^^,^^34^^,^^37^ As for data from the study by Smid et al^17^ in our previous review, the results of the study by Skrunes et al^33^ represent a pool of data from patients treated with agalsidase beta, switched from beta to alfa, and switched back from alfa to beta, and therefore were not included in the analysis of some parameters. Furthermore, Table 2 reports data of male patients of the included studies.

**Table 1:** Main characteristics of eligible studies

Study	No. of Patients	Control Group	Gender (M/F)	Age	GLA Variant	Preshortage ERT Age	Switch Period	Reswitch Period	Outcomes	Results
Lin et al^16^	9	No	9/0	43.4 ± 16.7 y^a^	• 3 classic • 6 late onset	26.5 ± 35.6 mo^a^	>1 year	NA	• Cardiac status: LVMI, SWT, LPWd • Renal function: UACr, eGFR • Plasma lyso-Gb3 • MSSI • Adverse events	• No change in all parameters • Improvement in LVMI
Smid et al^17^	20	No	NA	NA	NA	5.4 (0.7-88.6) y^b^	1.3 (0.9-1.5) y^b^	NA	• Changes in clinical events • Renal function: eGFR • Cardiac status: LVMI • QoL, pain • Plasma lyso-Gb3 • Adverse events	• No change in all parameters • Increase of lyso-Gb3 levels
Lenders et al^19^	37	No	20/17	46.1 ± 13.9 y^a^	NA	44.2 ± 26.3 mo^a^	2 y	NA	• Changes in clinical events • Cardiac status: LVMI, LVPWd, SWT, EF, ECG • Renal function: eGFR, UACr • Neurologic status: clinical examination and interview on TIA/stroke • FD-related symptoms • MSSI	• Worsening in eGFR • Worsening of MSSI • No change in other parameters
Tsuboi and Yamamoto^18^	11	No	4/7	47.3 ± 15.0 y^a^	All classic	47.8 ± 19.4 mo^a^	3 y	NA	• Changes in clinical events • Cardiac status: LVMI, LVPWd, SWT, EF, ECG • Renal function: GFR, UACr • Pain, QoL, MSSI • Plasma lyso-Gb3 • Antibody analysis • Adverse events	• No change in all parameters • Improvement in LVMI, SWT, LVPWd, EF • One patient positive for ADA antiagalsidase beta
Pisani et al^20^	10	No	7/3	44.3 ± 5.5 y^a^	All classic	>48 mo	20 mo	NA	• Changes in clinical events • Cardiac status: LVMI, LVPWd, EF, ECG • Renal function: eGFR, UACr • Health status, QoL, pain • Adverse events	• No change in all parameters
Wakabayashi et al^21^	18	No	8/10	42.0 ± 16.9 y^a^	• 7 NA • 7 late onset • 4 classic	36.3 ± 17.1 mo^a^	2 y	NA	• Renal function: sCr, SUN, eGFR, UACr	• No change in all parameters
Goker-Alpan et al^22^	71	No	40/31	46.4 (5-84) y^b^	NA	4.6 (0.3-12.2) y^b^	2 y	NA	• Changes in clinical events • Cardiac status: LVMI, MFS • Renal function: eGFR, UACr • Adverse events	• No change in all parameters
West et al^23^	36	No	24/12	50.2 ± 12.7 y^a^	NA	70.3 ± 25.1 mo^a^	15.1 ± 1.9 mo^a^	NA	• Cardiac status: LVPWd, LVMI, SWT, BP, NYHA grade • Renal function: eGFR, CKD stage, UACr • MSSI	• No change in all parameters • Worsening in SWT
Reidt et al^24^	5	Yes	5/0	NA	NA	57.6 ± 5.4 mo^a^	2 y	NA	• Changes in clinical events • Cardiac status: LVMI, LVPWd, SWT, EF, ECG; • Renal function: sCr, eGFR, UACr • Pain, QoL, MSSI • Lyso-Gb3	• No change in all parameters • Worsening in UACr and sCr in 2 patients
Lenders et al^34^	61 (39 reswitch, 22 switch)	Yes	22/17 reswitch 16/6 switch	48 ± 12 y reswitch^a^ 40 ± 14 y switch^a^	NA	55 ± 29 mo^a^ (reswitched patients) 47 ± 37 mo^a^ (switched patients)	39 ± 14 mo^a^ (reswitched patients) 82 ± 22 mo^a^ (switched patients)	55 ± 19 mo^a^	• Changes in clinical events • 4Cardiac status: SWT, LVPWd, EF • Renal function: eGFR, UACr • FD-related symptoms • MSSI • Plasma lyso-Gb3	• Worsening of eGFR: in male switched patients after the switch and in reswitched patients, after the reswitch • Decrease of lyso-Gb3 levels after switch-back to agalsidase beta • Increase of MSSI in both groups • Worsening of pain after the switch to agalsidase alfa and amelioration after switch-back to agalsidase beta • No change in other evaluated parameters
Politei et al^35^	2	No	2/0	43.0 ± 15.6 y^a^	All classic	> 4 y	>14 mo	NA	• Cardiac status: SWT, LVPWd, LVMI • Renal function: eGFR, proteinuria • Neurologic status: MRI • Pain	• Worsening of eGFR in 1 patient • Doubling of proteinuria in one patient • Worsening of cardiac parameters in 1 patient • Worsening of pain after switch and amelioration after reswitch • No change in other parameters
Limgala et al^36^	15	Yes	6/9	33.1 ± 19.1 y^a^	All classic	NA	NA	12 mo	• Antibody analysis: ADA antialfa, NAb • Immunophenotyping • Plasma and urine lyso-Gb3	• Development of ADA in 6 male patients during both ERT periods (NAb in 3 patients), none seroconversion • Same immune abnormalities with both ERT • Decrease of plasma and urine lyso-Gb3 levels after switch to agalsidase beta • No change in other parameters
Goker-Alpan et al^37^ (INFORM Study)	15 (9 switch beta-alfa-beta, 6 switch alfa-beta)	No	15/0	28.5 ± 16.1 y^a^	• 12 classic • 2 late onset • 1 NA	0.3-7 (mean = 3.5) y	1.6-14 (mean = 3.7) y	6 mo	• Plasma lyso-Gb • Plasma and urine Gb3 • Antibody analysis: ADA, NAb • Adverse events	• Decrease of plasma Gb3 and lyso-Gb3 levels after switch to agalsidase beta • Development of ADA for both ERT in 8 patients (NAb in 1 patient) during agalsidase alfa therapy, 1 seronegative patient seroconverted after switch to agalsidase beta • No change in other parameters
Skrunes et al^33^	10 (pooled data of 2 patients stably on agalsidase β, 3 switched from β to α, 5 switched from α to β)	No	NA	NA	NA	NA	NA	NA	• Changes in clinical events • Renal function: eGFR, UACr • Change in kidney biopsies • Plasma lyso-Gb3 • Antibody analysis: NAb • General disease load: Fabry-DS3	• Renal function, plasma lyso-Gb3 and kidney biopsies not evaluable • 1 male patient positive for NAb during agalsidase alfa therapy, with doubling of titer after switch to beta • Worsening of Fabry-DS3 in 5/10 patients when agalsidase dose was reduced to <1.0 mg/kg/e.o.w. • No change in other parameters
Ripeau et al^32^	33	No	23/10	28.7 ± 2.1 y^a^	NA	12 ± 2 mo^a^	2 y	NA	• Cardiac status: SWT, LVPW, LVMI • Renal function: eGFR, proteinuria • FD-related symptoms • MSSI • Adverse events	• No change in all parameters • Worsening of BPI (22%) • Worsening of physical functioning domain of SF-36

*ADA*, antiagalsidase alfa antibodies; *BPI*, brief pain inventory; *CKD*, chronic kidney disease; *ECG*, electrocardiogram; *EF*, ejection fraction; *eGFR*, estimated glomerular filtration rate; *ERT*, enzyme replacement therapy; *F*, female; *Fabry-DS3*, Fabry disease severity scoring system; *FD*, Fabry disease; *LVMI*, left ventricular mass index; *LVPWd*, left ventricular posterior wall diameter; *Lyso-Gb3*, lyso-globotriasolylceramide 3; *M*, male; *MFS*, midwall fractional shortening; *MRI*, magnetic resonance imaging; *MSSI*, Mainz Severity Score Index; *NA*, not applicable; *NAb*, neutralizing antibodies; *QoL*, quality of life; *sCr*, serum creatinine; *SF-36*, Short Form 36; *SUN*, serum urea nitrogen; *SWT*; septal wall thickness; *TIA*; transient ischemic attack; *UACr*, urine albumin-to-creatinine ratio.

a Mean ± SD.

b Median and interquartile range.

**Table 2:** Main characteristics of male patients in eligible studies

Study	No. of Male Patients	Age	GLA Variant	Results
Lin et al^16^	9	43.4 ± 16.7 y^a^	• 3 classic • 6 late onset	• No change in all parameters • Improvement in LVMI
Smid et al^17^	NA	NA	NA	NA
Lenders et al^19^	20	NA	NA	• No reported difference between genders for all parameters (Table 1)
Tsuboi and Yamamoto^18^	4	38.2 ± 6.2 y^a^	All classic	• One male patient positive for ADA antiagalsidase beta • No reported difference between genders for other parameters (Table 1)
Pisani et al^20^	7	46.0 ± 5.7 y^a^	All classic	• No reported difference between genders for all parameters (Table 1)
Wakabayashi et al^21^	8	32.2 ± 13.4 y^a^	• 3 NA • 3 classic • 2 late onset	• No reported difference between genders for all parameters (Table 1)
Goker-Alpan et al^22^	40	40 (56.3) y^b^	NA	• Reduction of urine Gb3 levels at month 12 in males • No reported differences between genders for other parameters (Table 1)
West et al^23^	24	NA	NA	• No reported difference between genders for all parameters (Table 1)
Reidt et al^24^	5	NA	NA	• No change in all parameters • Worsening in UACr and sCr in 2 patients
Lenders et al^34^	22 reswitch, 16 switch	NA	NA	• Worsening of eGFR: in male switched patients after the switch, and in reswitched patients after the reswitch • No reported difference between genders for other parameters (Table 1)
Politei et al^35^	2	43.15 ± 15.6 y^a^	All classic	• Worsening of eGFR in 1 patient • Doubling of proteinuria in 1 patient • Worsening of cardiac parameters in 1 patient • Worsening of pain after switch and amelioration after reswitch • No reported difference between genders for other parameters (Table 1)
Limgala et al^36^	6	35.0 ± 17.7 y^a^	All classic	• Development of ADA in all male patients during both ERT periods (NAb in 3 patients) • No reported difference between genders for other parameters (Table 1)
Goker-Alpan et al^37^ (INFORM Study)	15 (9 switch beta-alfa-beta, 6 switch alfa-beta)	28.5 ± 16.1 y^a^	• 12 classic • 2 late onset • 1 NA	• Decrease of plasma Gb3 and lyso-Gb3 levels after switch to agalsidase beta • Development of ADA for both ERT in 8 patients (NAb in 1 patient) during agalsidase alfa therapy; 1 seronegative patient seroconverted after switch to agalsidase beta
Skrunes et al^33^	NA	NA	NA	• 1 male patient positive for NAb during agalsidase alfa therapy, with doubling of titer after switch to beta • No reported difference between genders for all parameters (Table 1)
Ripeau et al^32^	23	29.4 ± 2.3 y^a^	NA	No reported difference between genders for all parameters (Table 1)

*ADA*, antiagalsidase alfa antibodies; *eGFR*, estimated glomerular filtration rate; *ERT*, enzyme replacement therapy; *LVMI*, left ventricular mass index; *Lyso-Gb3*, lyso-globotriasolylceramide 3; *NA*, not applicable; *NAb*, neutralizing antibodies; *sCr*, serum creatinine; *UACr*, urine albumin-to-creatinine ratio.

a Mean ± SD.

b Median and interquartile range.

### Death and clinical events

Nine studies explored these outcomes.^17^, ^18^, ^19^, ^20^^,^^22^^,^^24^^,^^33^, ^34^, ^35^

Regarding the outcome death, 8 events were reported in a total of 234 analyzed patients (3.4%): 1 death caused by cardiac arrest was described by Goker-Alpan et al^22^ and 7 deaths were reported by Lenders et al^34^ in a total population of 68 analyzed patients: 4 patients (mean age 61 years) died about 4.75 years after the switch (15.4% of switched patients); of these, 1 female died of cardiac decompensations, whereas causes of death were not available for the remaining 3 males. Moreover, other 3 patients (mean age 60 years) died about 5.0 years after reswitching to agalsidase beta (7.1% of reswitched patients): 1 female died of cancer, a male of sudden cardiac death, and the death cause of an additional female was unknown. However, authors reported no difference in the increased risk of death in any of the treatment groups.^34^

Regarding the outcome clinical events, the 9 studies reported 25 events in a total population of 227 patients (11.0%). More specifically, the authors described 2 not specified events,^17^ 5 cases of transient ischemic attack/stroke,^19^^,^^22^^,^^34^ an atrial fibrillation,^18^ 1 hemodialysis initiation,^19^ 2 renal transplantations,^34^ 9 implantations of a pacemaker/cardioverter-defibrillator,^19^^,^^34^ and 2 cases of worsening of renal function.^33^^,^^35^

Finally, Lenders et al^34^ reported 3 clinical events after reswitching from agalsidase alfa to beta (all implantations of pacemaker/cardioverter-defibrillator).

### Changes in organ function or structure

These changes were categorized as cardiac, renal, and neurologic.

Ten papers reported cardiac changes after switching therapy,^16^, ^17^, ^18^, ^19^, ^20^^,^^22^^,^^23^^,^^32^^,^^34^^,^^35^ with a total of 254 analyzed patients. As reported in the previous review, data in the study by Smid et al^17^ were reported as pooled data and were not included. Data were very heterogeneous; indeed, septum wall thickness ameliorated in 1 paper,^18^ unchanged in 3 studies,^19^^,^^32^^,^^34^ or even significantly worsened after switch.^23^ Similarly, 2 papers reported a significant improvement in left ventricular mass index,^16^^,^^18^ whereas the remaining ones did not report any change.^20^^,^^22^^,^^23^^,^^32^ Midwall fractional shortening,^22^ ejection fraction,^19^^,^^20^^,^^34^ and left ventricular posterior wall diameter^20^^,^^23^^,^^32^^,^^34^ remained unchanged, whereas an improvement in left ventricular posterior wall diameter (*P* = .00236) with a worsening in ejection fraction (*P* = .0340) was reported by Tsuboi and Yamamoto^18^ 3 years after the switch. Furthermore, 4 publications did not report any change in electrocardiogram data.^18^^,^^19^^,^^32^^,^^34^ Finally, Politei et al^35^ reported the case of 1 patient with FD with left ventriicular hypertrophy, who remained stable during therapy with agalsidase beta and showed a worsening after the switch to alfa.

In total, 13 papers reported the effects of switch on renal function.^16^, ^17^, ^18^, ^19^, ^20^, ^21^, ^22^, ^23^, ^24^^,^^32^, ^33^, ^34^, ^35^ For example, data in Smid et al^17^ from the paper by Skrunes et al^33^ were reported as pooled data, which included either patients always treated with agalsidase beta or switched to agalsidase alfa at time of beta shortage, and switched from agalsidase alfa to beta because of disease progression or suboptimal treatment effect, and were not evaluated. However, the authors described the interesting case of a male patient with FD with significant fall in eGFR after switching from agalsidase beta to alfa (>5 mL/min per 1.73 m^2^/y) followed by a significant improvement after reinstating agalsidase beta.^33^ No change was reported in 9 studies in urine albumin-to-creatinine ratio^16^^,^^18^, ^19^, ^20^, ^21^, ^22^, ^23^^,^^32^^,^^34^ and in 8 studies in eGFR.^16^^,^^18^^,^^20^, ^21^, ^22^, ^23^, ^24^^,^^32^ In their earlier study, Lenders et al^19^ reported a significant worsening of cystatine-C–based and creatinine/cystatine-C–based eGFR after the switch, whereas creatinine-based eGFR remained stable. The results of the more recent paper by Lenders et al,^34^ showed a slight nonsignificant loss of eGFR in both switched and reswitched patients; however, when stratified for sex, the loss of eGFR became significant in male switched patients (−2.9 ± 2.7 mL/min per 1.73 m^2^/y, *P* < .001). Moreover, both switched and reswitched patients suffered from a significant eGFR loss in the longer follow-up period (44 ± 13 months after the first follow-up and 55 ± 19 months after the reswitch, respectively).^34^

Finally, Politei et al^35^ reported a deterioration in renal function after the switch in his 2 patients: the first patient showed doubling of proteinuria 34 months after switch to agalsidase alfa with a stable eGFR; the other patient, who had a significant loss in eGFR of 6 mL/min over 4 years of therapy with agalsidase beta (progressing from chronic kidney disease stage 2 to 3), reported unchanged proteinuria and a significant progression of eGFR loss to stage 5 in 14 months after the switch to alfa, for which he started dialysis.^35^

Neurologic changes were reported only in 2 studies,^19^^,^^35^ in which all performed evaluations were reported to remain stable.

### Change in FD-related symptoms

The study by Skrunes et al^33^ reported general disease load using the FD severity scoring system,^42^ but, as for other endpoints, it was impossible to evaluate the effects of switch on this parameter. However, an increase in FD severity scores was reported in 5 of 10 patients when agalsidase dose was reduced to <1.0 mg/kg/e.o.w.^33^

Six studies reported on MSSI^16^^,^^18^^,^^19^^,^^23^^,^^32^^,^^34^: 3 studies did not report any change after the switch^19^, ^24^, ^33^ (*N* = 70 patients); Lin et al^16^ observed a stability of the disease at 1 year, with mild improvements in cardiac or renal scores in 3 patients out of 9, and a slight worsening in general score in 1 subject; a worsened MSSI, conversely, was reported by Lenders et al^19^ in their earlier study (*P* = .001, *n* = 37 patients), and later confirmed in their more recent paper in both switched and reswitched patients over time (*n* = 61 patients), with no difference between groups.^34^

Seven papers reported on pain.^17^, ^18^, ^19^, ^20^^,^^32^^,^^34^^,^^35^ As for the other parameters, data in the paper by Smid et al^17^ were not included in the analysis also for pain; 3 studies reported no difference in pain severity after switching^18^, ^19^, ^20^; moreover, Lenders et al^19^ first reported that switched patients had less frequent pain attacks than patients on agalsidase beta, despite a greater abdominal pain. Conversely, in their more recent paper, the authors reported that reswitched patients presented a significant increase of pain after switching to agalsidase alfa, which was stablished after switch-back to beta, whereas in switched patients, pain did not increase.^34^ A significant increase of 22% in BPI was also observed after 2 years of switching to agalsidase alfa in the cohort of 33 patients with FD described by Ripeau et al.^32^ Finally, the 2 patients described by Politei et al ^35^ reported a favorable change in pain during agalsidase beta therapy, a return of distal pain after switching to alfa and an amelioration after returning to agalsidase beta therapy.

In the 4 studies reporting on changes in QoL,^17^^,^^18^^,^^20^^,^^32^ the different scales of QoL remained unchanged before and after switching,^18^^,^^20^^,^^32^ except for a worsening of physical functioning domain of SF-36 reported by Ripeau et al^32^ in 33 patients with FD after 2 years of switching.

Only Lenders et al^19^^,^^34^ reported, in their 2 papers, that modifications in other symptoms such as gastrointestinal symptoms, diarrhea, tinnitus, or ability to sweat were not evaluated after the switch.

### Change in lyso-Gb3 plasma concentration

Six studies reported on changes in lyso-globotriaosylceramide (lyso-Gb3) concentration after switching from agalsidase beta to alfa,^16^, ^17^, ^18^^,^^22^^,^^33^^,^^34^ whereas 2 studies reported data after switching from agalsidase alfa to beta.^36^^,^^37^

Smid et al^17^ reported an increase in lyso-Gb3 in male patients switched to agalsidase alfa after 12 months, even if it should be considered that in all these patients, a 6-month dose reduction to 25% of the initial dose of agalsidase beta before the shift was performed. In addition, the authors reported no significant difference in the increase of plasma lysoGb3 levels between males with and without neutralizing antibodies to agalsidase beta.^17^

On the contrary, 4 papers reported no change in plasma lysoGb3 levels after the switch.^16^^,^^18^^,^^22^^,^^34^ Furthermore, Lenders et al^34^ also reported a significant decrease in lyso-Gb3 levels after reswitch from agalsidase alfa to beta. Interestingly, to exclude a solely neutralizing effect of ADA on lyso-Gb3 after the reswitch, the authors further analyzed a subgroup of males with available lyso-Gb3 and ADA status. The results of such analysis showed that the reduction in lyso-Gb3 observed after the switch-back was greater in subjects with neutralizing ADAs.^34^

Data by Skrunes et al^33^ were reported as pooled data and were not evaluable; however, lyso-Gb3 levels were reported to correlate with cumulative agalsidase dose received in males, and the authors showed that they were significantly lower in patients receiving higher than in the patients receiving lower agalsidase dose.

In addition to Lenders et al,^34^ other 2 studies investigated the changes in lyso-Gb3 levels after shift of therapy^36^^,^^37^; in both the papers, a significant decrease after the change of therapy was reported: the reduction was observed within 2 months and continued to further decrease over time at month 4 and 6 in INFORM study^37^ and 1 year after switching to agalsidase beta by Limgala et al, ^36^ despite their levels remained above their reference values.

### Antibodies

Four studies explored this outcome^18^^,^^32^^,^^36^^,^^37^ with a total of 51 patients evaluated.

Tsuboi and Yamamoto^18^ reported only 1 treatment related allergic reaction to agalsidase beta in a patient positive for antibodies, antiagalsidase beta, that disappeared after switch to alfa. On the contrary, all patients were negative for antiagalsidase alfa antibody before switching, including the patient with antiagalsidase beta antibody and remained negative after 36 months.^18^

Skrunes et al^33^ reported that no patient developed inhibitory antibodies, except 1 patient who became positive to neutralizing ADA after 10 years of agalsidase alfa and reported a transient doubling of the titer after switch to agalsidase beta.

The INFORM study reported, in a cohort of 15 patients on agalsidase alfa (9 switched from beta and 6 naϊve), 8 patients positive for ADA for both ERT; although 3 of these patients had never received agalsidase beta, 7 patients were seronegative and 4 of these received both agalsidase alfa and beta, and neutralizing antibodies were found in only 1 patient (naϊve to ERT). No patient was positive for antibodies against one ERT and negative for the other; furthermore, only 1 seronegative patient seroconverted after switch to agalsidase beta.^37^ Similarly, in a group of 15 patients switched from agalsidase alfa to beta, Limgala et al^36^ reported 6 patients positive for antialfa ADA during both ERT periods; of these, 3 subjects were positive for neutralizing antibodies. Furthermore, no patient seroconverted after the switch.

### AEs

Six papers (*n* = 191 subjects) evaluated this outcome.^16^, ^17^, ^18^^,^^20^^,^^22^^,^^32^ A total of 30 patients underwent at least 1 AE after the shift of therapy (15.7% of subjects). In detail, 6 mild infusion adverse reactions were reported by Pisani et al,^20^ 1 by Smid et al,^17^ and 2 by Lin et al.^16^ Goker-Alpan et al^22^ reported serious AEs, though unspecified, and 21 patients with AE after the switch, whereas no AE was observed by Tsuboi and Yamamoto^18^ and Ripeau et al.^32^

On the contrary, the INFORM study reported a nonserious AE experienced by only 1 of the 15 patients (7%) shifted to agalsidase beta positive for antiagalsidase antibodies. The AE occurred after the first agalsidase beta infusion, resolved after 2 days, and the patient continued therapy with no further AEs.^37^

## Discussion

In 2016, a meta-analysis with a complete review on the effects of switch from agalsidase beta to alfa in FD was published. The authors analyzed all the literature until 2015 (9 studies and 218 patients) and concluded that the shift to agalsidase alfa did not negatively influence the course of FD in the short term.

In this paper, we performed a re-evaluation of the existing literature with an update of the systematic review, mainly focusing on the effects of the shift to agalsidase alfa in the long term, with a mention on the effects of reswitch to agalsidase beta. The updated review identified 6 new papers (including 136 subjects) published on this topic since September 2015; consequently, a total of 15 publications were included with a total of 353 patients. The results of this updated review confirmed some points in the prior analysis and gave some new important information.

At first, compared with the previous review, the incidence of clinical events resulted after the switch increased: compared with 1 death (0.6%) and 7 events reported in 154 patients (4.5%) in the previous paper, the updated review report 7 deaths (3.1%) and a total of 25 events (11%) in 227 patients. The observed increased number of clinical events in comparison with that in the previous review could reflect the longer observation period.

Data on cardiac function showed a stability in most of the examined parameters after the switch; in particular, only study by Politei et al^35^ described the case of 1 patient treated with agalsidase beta, with stable left ventriicular hypertrophy that frankly worsened after the switch to agalsidase alfa.^35^

About renal function, a worsening of eGFR after the switch was reported in 4 papers.^19^^,^^33^, ^34^, ^35^ Skrunes et al^33^ also described the case of a significant eGFR loss after switch and improved after shift back to agalsidase beta.

Although most of the questionnaires remain largely subjective about FD-related symptoms because of self-reported measures, no peculiar difference emerged between the 2 formulations in MSSI score, QoL, and other FD-related symptoms, which were stable in most papers, with the exception of both the papers by Lenders et al,^19^^,^^34^ who reported worsening of MSSI in both switched and reswitched patients. Furthermore, in more recent studies, pain worsened after shifting to agalsidase alfa,^32^^,^^34^^,^^35^ with amelioration after reswitch to agalsidase beta.^34^^,^^35^

Conversely, plasma lyso-Gb3 levels represent an objective measure of disease progression. Most studies reporting on this outcome supported ERT dosage effects on lyso-Gb3 levels.^17^^,^^33^^,^^34^^,^^36^^,^^37^ At first, Smid et al^17^ showed an increase in lyso-Gb3 levels in switched males previously receiving reduced doses of agalsidase beta. This observation supports the earlier findings that the critical determinant of the biochemical response is the dose and not the type of enzyme formulation because such increase could be attributed to the reduced ERT dose. Moreover, Skrunes et al^33^ reported that subjects treated with higher ERT dose had lyso-Gb3 levels significantly lower than the patients treated with lower dose, showing a correlation between plasma lyso-Gb3 levels and the cumulative agalsidase dose received. Again, 3 papers reported a significant decrease in lyso-Gb3 levels after reswitch from agalsidase alfa to beta.^34^^,^^36^^,^^37^ In line with these observations, a dose-depending clearing of podocyte Gb3 has previously been shown in classic Fabry subjects receiving ERT: the reduction of podocyte Gb3 was found to increase with increasing agalsidase dose.^31^^,^^43^

Agalsidase alfa and beta have epitopes in common, and such structural similarities suggest similar immunogenic profiles, as shown by the results of the 4 papers reporting data on antibodies.^18^^,^^33^^,^^36^^,^^37^ Moreover, some patients receiving agalsidase alfa and never exposed to beta were found positive for agalsidase beta antibody,^37^ whereas only 1 patient seroconverted after the shift.^37^

The limitations of this study mainly reflect the characteristics of rare genetic diseases, such as the heterogeneity of study population; small number of patients and, consequently, the small sample size of the studies, which may preclude observing differences between the study groups; short observation period; and slow progression of FD, which could account for the low number of reported clinical events. In addition, the clinical presentation of FD is highly heterogeneous, and the timing of symptom onset is highly variable.

Furthermore, the main limitation of this study is the lack of data available for a meta-analysis.

In conclusion, in accordance with the previous review, our updated data suggest that a switch from agalsidase beta to alfa was generally safe. However, some of the new papers reported, after the switch, a significant loss of renal function, which was not reversed even by higher dosages, and an increase in lyso-Gb3 levels. On the contrary, a shift from agalsidase alfa to beta lead to a decrease in lyso-Gb3 levels. These findings emphasize the importance of dose in the treatment of FD and recommend that patients who received dose-reduced ERT be monitored through an intensified surveillance, including lyso-Gb3 levels every 6 months.

## Data availability statement

The authors declare that all data are available and will be sent individually upon request.

## Funding

The authors did not receive financial support for this article.

## Author Information

Conceptualization: E.R., C.G., A.P.; Data curation: C.G., E.R.; Formal analysis: C.G.; Funding acquisition: F.R.C.; Investigation: E.R., C.G.; Methodology: C.G.; Project administration: A.P.; Resources: E.R.; Software: T.D.R., M.I., G.I.; Supervision: A.P., E.R.; Validation: A.P.; Visualization: I.C., P.B.; Writing-original draft: E.R.; Writing-review and editing: A.P., E.R.

## Conflict of Interest

AP received travel expenses and grants from 10.13039/100007723Takeda Shire, 10.13039/100013995Sanofi Genzyme, 10.13039/100015362Amicus, and 10.13039/100019719Chiesi. All other authors have no conflict of interest.
